# Data assimilation for the heat equation using stabilized finite element methods

**DOI:** 10.1007/s00211-018-0949-3

**Published:** 2018-02-10

**Authors:** Erik Burman, Lauri Oksanen

**Affiliations:** Department of Mathematics, University College London, Gower Street, London, WC1E 6BT UK

**Keywords:** 65M12, 65M15, 65M30, 65M32

## Abstract

We consider data assimilation for the heat equation using a finite element space semi-discretization. The approach is optimization based, but the design of regularization operators and parameters rely on techniques from the theory of stabilized finite elements. The space semi-discretized system is shown to admit a unique solution. Combining sharp estimates of the numerical stability of the discrete scheme and conditional stability estimates of the ill-posed continuous pde-model we then derive error estimates that reflect the approximation order of the finite element space and the stability of the continuous model. Two different data assimilation situations with different stability properties are considered to illustrate the framework. Full detail on how to adapt known stability estimates for the continuous model to work with the numerical analysis framework is given in “Appendix”.

## Introduction

We consider two data assimilation problems for the heat equation

1 $$\begin{aligned} \partial _t u - \Delta u = f, \quad \text { in } (0,T) \times \Omega , \end{aligned}$$

where $T > 0$ and $\Omega \subset \mathbb R^n$ is open. Let $\omega , B \subset \Omega$ be open and let $0< T_1 < T_2 \le T$. Both the data assimilation problems are of the following general form: determine the restriction $u|_{(T_1, T_2) \times B}$ of a solution to the heat equation (1) given *f* and the restriction $u|_{(0,T) \times \omega }$. The crucial difference between the two problems is that in the first problem we do not make any assumptions on the boundary condition satisfied by *u*, whereas in the second problem we assume that it satisfies the lateral Dirichlet boundary condition $u|_{(0,T) \times \partial \Omega } = 0$. We emphasize that, in both the problems, no information on the initial condition satisfied by *u* is assumed to be known.

For the first problem we assume the following geometry,

1. *B* is connected, $\omega \subset B$, and the closure $\overline{B}$ is compact and contained in $\Omega$, and in the second case we may choose $B = \Omega$ while assuming that
2. $\overline{\Omega }$ is a compact, convex, polyhedral domain.

The first problem is conditionally Hölder stable, and this stability is optimal. More precisely, the following continuum stability estimate holds.

### Theorem 1

Let $\omega \subset \Omega$ be open and non-empty, and let $0< T_1< T_2 < T$. Suppose that an open set $B \subset \Omega$ satisfies (A1). Then there are $C > 0$ and $\kappa \in (0,1)$ such that for all *u* in the space

2 $$\begin{aligned} H^1(0,T; H^{-1}(\Omega )) \cap L^2(0,T; H^1(\Omega )), \end{aligned}$$

it holds that

$$\begin{aligned}&\Vert u\Vert _{L^2(T_1, T_2; H^1(B))} \le C (\Vert u\Vert _{L^2((0, T) \times \omega )} \\&\quad + \Vert L u\Vert _{(0,-1)})^\kappa (\Vert u\Vert _{L^2((0, T) \times \Omega )} + \Vert L u\Vert _{(0,-1)})^{1-\kappa }, \end{aligned}$$

where $$L = \partial _t - \Delta $$ and $$\Vert \cdot \Vert _{(0,-1)} = \Vert \cdot \Vert _{L^2(0, T; H^{-1}(\Omega ))}$$.

In Theorem 1, the factor with the power $\kappa$ can be viewed as the size of the data

3 $$\begin{aligned} q = u|_{(0, T) \times \omega }, \quad f = Lu, \end{aligned}$$

and the norm $\Vert u\Vert _{L^2((0, T) \times \Omega )}$ as an apriori bound for the unknown *u*. Let us also emphasize that the assumption $\overline{B} \subset \Omega$ is essential, indeed, if $\overline{B} \cap \partial \Omega \ne \emptyset$ then the optimal stability is conditionally logarithmic. The second problem is stable.

### Theorem 2

Let $\omega \subset \Omega$ be open and non-empty, and let $0< T_1 < T$. Suppose that (A2) holds. Then there is $C > 0$ such that for all *u* in the space

4 $$\begin{aligned} H^1(0,T; H^{-1}(\Omega )) \cap L^2(0,T; H_0^1(\Omega )), \end{aligned}$$

it holds that

$$\begin{aligned}&\Vert u\Vert _{C(T_1, T; L^2(\Omega ))} + \Vert u\Vert _{L^2(T_1, T; H^1(\Omega ))} + \Vert u\Vert _{H^1(T_1, T; H^{-1}(\Omega ))}\\&\quad \le C (\Vert u\Vert _{L^2((0, T) \times \omega )} + \Vert L u\Vert _{(0,-1)}). \end{aligned}$$

The estimates in Theorems 1 and 2 are tailored to work well with the finite element discretizations that are the main topic of the present paper. We review the literature on continuum stability estimates and give the proofs bridging the gap between the existing estimates and those in Theorems 1 and 2 in an “Appendix”.

In what follows, we call the two data assimilation problems the unstable and the stable problem, respectively. We use the shorthand notation

$$\begin{aligned} a(u,z) = (\nabla u, \nabla z),\quad G_f(u,z) = (\partial _t u, z) + a(u,z) - \left\langle f,z \right\rangle , \quad G = G_0, \end{aligned}$$

where $(\cdot ,\cdot )$ is the inner product of $L^2((0,T) \times \Omega )$ and $\left\langle \cdot ,\cdot \right\rangle$ is the dual pairing between $L^2(0,T; H^{-1}(\Omega ))$ and $L^2(0,T; H_0^1(\Omega ))$. Note that for $u \in H^1((0,T) \times \Omega )$, the equations

5 $$\begin{aligned} G_f(u,z) = 0, \quad z \in L^2(0,T; H_0^1(\Omega )), \end{aligned}$$

give the weak formulation of $\partial _t u- \Delta u = f$. Our approach to solve the data assimilation problem, in both the cases, is based on minimizing the Lagrangian functional

6 $$\begin{aligned} L_{q,f}(u,z) = \frac{1}{2} \Vert u - q\Vert _{\omega }^2 + \frac{1}{2} s(u,u) - \frac{1}{2} s^*(z,z) + G_f(u,z), \end{aligned}$$

where the data *q* and *f* are fixed. Here $\Vert \cdot \Vert _\omega$ is the norm of $L^2((0,T) \times \omega )$, and *s* and $s^*$ are symmetric bilinear forms, that are chosen differently in the two cases, and that we call the primal and dual stabilizers, respectively. Note that minimizing $L_{q,f}$ can be seen as fitting $u|_{(0,T) \times \omega }$ to the data *q* under the constraint (5), *z* can be interpreted as a Lagrange multiplier, and *s* / 2 and $s^*/2$ as regularizing penalty terms.

In this paper we consider only semi-discretizations, that is, we minimize $L_{q,f}$ on a scale of spaces that are discrete in the spatial variable but not in the time variable. The spatial mesh size $h > 0$ will be the only parameter controlling the convergence of the approximation, and we use piecewise affine finite elements.

An important feature of the present work is that the choice of the regularizing terms is driven by the analysis and designed to give error estimates that reflect the approximation properties of the finite element space and the stability of the continuous model. In particular, when the continuous model is Lipschitz stable, we obtain optimal error estimates in the usual sense in numerical analysis, that is, the estimates are optimal compared to interpolation of the exact solution. This is the case for the second model problem introduced above. The regularization is constructed on the discrete level, that is, *s* and $s^*$ are defined on the semi-discrete spaces, and in some cases, they may not even make sense on the continuous level.

We show how the different stability properties of the two model problems lead to different regularization operators. The choice for a given problem is not unique, and the design of regularizing terms leading to optimal estimates can also be driven by computational considerations, such as computational cost or couplings in the discrete formulations. Therefore we present an abstract framework for the design of regularization operators. When measurement errors are present we also show how to quantify the damping of perturbations.

For the unstable problem, under suitable choices of the semi-discrete spaces and the regularization, we show that (6) has a unique minimizer $(u_h, z_h)$, $h > 0$, satisfying the following convergence rate

7 $$\begin{aligned} \Vert u_h - u\Vert _{L^2(T_1, T_2; H^1(B))}&\le C h^\kappa (\Vert u\Vert _{*} + \Vert f\Vert ), \end{aligned}$$

where *u* is the unique solution of the continuum data assimilation problem, $\kappa \in (0,1)$ is the constant in Theorem 1, and the norms on the right-hand side capture the assumed apriori smoothness, see Theorem 3 below for the precise formulation. For the stable problem, we establish an analogous result but with $\kappa = 1$, that is, the convergence rate reflects now the stability estimate in Theorem 2. Moreover, in the stable case, we can replace the norm on the left-hand side of (7) with the stronger norm in Theorem 2, see Theorem 6 below for the precise formulation.

We consider the time discretization of the stable problem in a separate paper [10]. The approach there is an extension of the strategy in the present paper: a Lagrangian of the form (6) is first discretized and then the stabilizers are chosen in the discrete spaces. The main difference is that an additional stabilization in time is required. In [10] we describe also two computational implementations of the method: one using an off-the-self linear solver, and the other using an iterative scheme that is based on the interpretation of the Euler–Lagrange equation for (6) as a system of two coupled heat equations. Let us also remark that the convergence analysis of our method is unchanged if the stabilizers in (6) are rescaled. We provide a computational study of such rescaling in [10].

### Previous literature

The classical approach to data assimilation and inverse problems is to introduce Tikhonov regularization on the continuous level [2, 15, 26] and then discretize the well-posed regularized model. The use of Carleman estimates for uniqueness of inverse problems and the quantification of stability was first proposed in [7]. The use of conditional stability to choose a Tikhonov regularization parameter has been explored in [13]. Nevertheless a common feature of all methods where regularization takes place on the continuous level is that the accuracy of the reconstruction will be determined by the regularization error, leaving no room to exploit the interplay between discretization and regularization.

The method we propose is an instance of the so-called 4DVAR method [21]. The analysis in the present paper, which draws on previous ideas in the stationary case [8, 9, 11], is to our best knowledge the first complete numerical analysis of a 4DVAR type method. The main characteristic of our approach is to separate the numerical stability and the stability of the continuous model, and use the continuous stability estimate in the perturbation analysis.

In contrast, another recent approach to parabolic data assimilation problems is to derive Carleman estimates directly on the discrete scheme [3, 4], which may then be used for convergence analysis. Other methods for data assimilation include the so called back and forth nudging [1] and methods using null-controllability [18, 23]. In the former case, forward and backward solves are combined with filtering techniques (or penalty) to drive the approximate solution close to data, and the latter case leads to an approximation algorithm which uses auxiliary optimal control problems. For another example of a finite element method for identification of the initial data using Tikhonov regularization see [12] and for a discussion of different approaches to numerical approximation of control and inverse problems we refer to the monograph [17, Chapter 5].

## Spatial discretization

We begin by observing that we may assume without loss of generality that $\Omega \subset \mathbb R^n$ is a compact and connected polyhedral domain also in the case (A1). Indeed, we can choose a compact and connected polygonal domain $\tilde{\Omega }\subset \Omega$ such that $\overline{B} \subset \tilde{\Omega }$ and then replace $\Omega$ by $\tilde{\Omega }$.

Consider a family $\mathcal T = \{\mathcal {T}_h;\ h > 0\}$ of triangulations of $\Omega$ consisting of simplices such that the intersection of any two distinct simplices is either a common vertex, a common edge or a common face. Moreover, assume that the family $\mathcal {T}$ is quasi uniform, see e.g. [16, Def. 1.140], and indexed by

$$\begin{aligned} h = \max _{K \in \mathcal {T}_h} \text{ diam }(K). \end{aligned}$$

The family $\mathcal {T}$ satisfies the following trace inequality, see e.g. [6, Eq. 10.3.9],

8 $$\begin{aligned} \Vert u\Vert _{L^2(\partial K)} \le C \left( h^{-\frac{1}{2}} \Vert u\Vert _{L^2(K)} + h^{\frac{1}{2}} \Vert \nabla u\Vert _{L^2(K)}\right) , \quad u \in H^1(K), \end{aligned}$$

where the constant *C* is independent of $K \in \mathcal T_h$ and $h>0$.

Let $V_h$ be the $H^1$-conformal approximation space based on the $\mathbb {P}_1$ finite element, that is,

9 $$\begin{aligned} V_h = \left\{ u \in C(\bar{\Omega }):\ u\vert _K \in \mathbb {P}_1(K),\ K \in \mathcal {T}_h\right\} , \end{aligned}$$

where $\mathbb {P}_1(K)$ denotes the set of polynomials of degree less than or equal to 1 on *K*. We recall that the family $\mathcal {T}$ satisfies the following discrete inverse inequality, see e.g. [16, Lem. 1.138],

10 $$\begin{aligned} \Vert \nabla u\Vert _{L^2(K)} \le C h^{-1} \Vert u\Vert _{L^2(K)} , \quad u \in \mathbb {P}_1(K), \end{aligned}$$

where the constant *C* is independent of $K \in \mathcal T_h$ and $h>0$.

We choose an interpolator

$$\begin{aligned} \pi _h : H^1(\Omega ) \rightarrow V_h, \quad h > 0, \end{aligned}$$

that satisfies the following stability and approximation properties

11 $$\begin{aligned} \Vert \pi _h u\Vert _{H^1(\Omega )}&\le C \Vert u\Vert _{H^{1}(\Omega )}, \qquad u\in H^{1}(\Omega ),\ h > 0,\ \end{aligned}$$

12 $$\begin{aligned} \Vert u - \pi _h u\Vert _{H^m(\Omega )}&\le C h^{k-m}\Vert u\Vert _{H^{k}(\Omega )}, \qquad u\in H^{k}(\Omega ),\ h > 0,\ \end{aligned}$$

where $k=1,2$ and $m = 0, k-1$, and that preserves vanishing Dirichlet boundary conditions, that is, using the notation $W_h = V_h \cap H_0^1(\Omega )$,

13 $$\begin{aligned} \pi _h : H_0^1(\Omega ) \rightarrow W_h, \quad h > 0. \end{aligned}$$

A possible choice is the Scott–Zhang interpolator [25].

### Spatial jump stabilizer

In the unstable case, the regularization will be based on the spatial jump stabilizer that we will introduce next. We denote by $\mathcal {F}_h$ the set of internal faces of $\mathcal {T}_h$, and define for $F \in \mathcal {F}_h$,

$$\begin{aligned} \llbracket n \cdot u\rrbracket _F&= n_1 \cdot u|_{K_1} + n_2 \cdot u|_{K_2}, \end{aligned}$$

where $K_1, K_2 \in \mathcal {T}_h$ are the two simplices satisfying $K_1 \cap K_2 = F$, and $n_j$ is the outward unit normal vector of $K_j$, $j=1,2$. We define the jump stabilizer

14 $$\begin{aligned} \mathcal J_h(u_h,u_h) = \sum _{F \in \mathcal {F}_h} \int _F h \llbracket n \cdot \nabla u_h\rrbracket _F^2 ~\text{ d }s, \quad u_h \in V_h, \end{aligned}$$

where $\text{ d }s$ is the Euclidean surface measure on *F*.

#### Lemma 1

(Discrete Poincaré inequality) There is $C > 0$ such that all $u_h \in V_h$, and $h > 0$ satisfy

15 $$\begin{aligned} \Vert u_h\Vert _{L^2(\Omega )} \le C h^{-1} \left( \mathcal J_h(u_h,u_h)^{1/2} + \Vert u_h\Vert _{L^2(\omega )}\right) . \end{aligned}$$

#### Proof

The original results on Poincaré inequalities for piecewise $H^1$-functions may be found in [5]. For a detailed proof of (15) see [11]. $\square$

#### Lemma 2

There is $C > 0$ such that all $u_h \in V_h$, $v \in H_0^1(\Omega )$, $w \in H^2(\Omega )$ and $h > 0$ satisfy

16 $$\begin{aligned} (\nabla u_h, \nabla v)_{L^2(\Omega )}&\le C \mathcal J_h(u_h,u_h)^{1/2} \left( h^{-1} \Vert v\Vert _{L^2(\Omega )} + \Vert v\Vert _{H^1(\Omega )} \right) , \end{aligned}$$

17 $$\begin{aligned} \mathcal J_h(\pi _h w, \pi _h w)&\le C h^2 \Vert w\Vert _{H^2(\Omega )}^2. \end{aligned}$$

#### Proof

Towards (16) we integrate by parts and recall that $u_h$ is an affine function on each element to obtain

18 $$\begin{aligned} (\nabla u_h, \nabla v)_{L^2(\Omega )} = \sum _{K \in \mathcal T_h} \int _K \nabla u_h \cdot \nabla v ~\text{ d }x = h^{-1/2} \sum _{F \in \mathcal F_h} \int _F h^{1/2} \llbracket n \cdot \nabla u_h\rrbracket _F\, v ~\text{ d }s. \end{aligned}$$

Thus by the Cauchy–Schwarz inequality

$$\begin{aligned} (\nabla u_h, \nabla v)_{L^2(\Omega )} \le h^{-1/2} \mathcal J_h(u_h,u_h)^{1/2} \left( \sum _{F \in \mathcal {F}_h} \int _F v^2 \text{ d }s \right) ^{1/2}, \end{aligned}$$

and the inequality (16) follows by applying (8) to the last factor.

Let us now turn to (17). As $\llbracket n \cdot \nabla w\rrbracket _F = 0$, we have using (8)

$$\begin{aligned}&\int _F h \llbracket n \cdot \nabla \pi _h w\rrbracket _F^2 ~\text{ d }s =h \int _F \llbracket n \cdot \nabla (\pi _h w - w)\rrbracket _F^2 ~\text{ d }s\\&\quad \le C \sum _{j=1,2} \left( \Vert n_j \cdot \nabla (\pi _h w - w)\Vert _{L^2(K_j)}^2 + h^2 \Vert n_j \cdot \nabla (\pi _h w - w)\Vert _{H^1(K_j)}^2\right) . \end{aligned}$$

Summing over all internal faces and observing that

$$\begin{aligned} \Vert \nabla (\pi _h w - w)\Vert _{H^1(K_j)}^2 = \Vert \nabla (\pi _h w - w)\Vert _{L^2(K_j)}^2+ \Vert \nabla w\Vert _{H^1(K_j)}^2, \end{aligned}$$

we obtain

$$\begin{aligned}&\sum _{F \in \mathcal {F}_h} \int _F h \llbracket n \cdot \nabla \pi _h w\rrbracket _F^2 ~\text{ d }s \le C \left( \Vert \nabla (\pi _h w - w)\Vert _{L^2(\Omega )}^2 + h^2 \Vert w\Vert ^2_{H^2(\Omega )}\right) . \end{aligned}$$

The first term on the right-hand side satisfies

$$\begin{aligned} \Vert \nabla (\pi _h w - w)\Vert _{L^2(\Omega )}^2 \le C h^2 \Vert w\Vert _{H^2(\Omega )}^2, \end{aligned}$$

by (12). $$\square $$

## The unstable problem

There are several possible choices for the primal and dual stabilizers *s* and $s^*$ in (6), and different choices lead to numerical methods that may differ in terms of practical performance. To illustrate the main ideas of our approach, we begin by considering a concrete choice of the stabilizers in Sect. 3.1, and give an abstract framework in Sect. 4.

In what follows, we use the shorthand notations $H^{(k,m)} = H^k(0,T; H^m(\Omega ))$,

$$\begin{aligned} \Vert u\Vert _{(k,m)} = \Vert u\Vert _{H^k(0,T; H^m(\Omega ))}, \quad k, m \in \mathbb R, \end{aligned}$$

and $H_0^{(k,m)} = H^{(k,m)} \cap L^2(0,T; H_0^1(\Omega ))$. We recall also that $\Vert u\Vert = \Vert u\Vert _{(0,0)}$ and that $\Vert u\Vert _\omega$ is the norm of $L^2((0,T) \times \omega )$.

### A model case

We choose the following primal and dual stabilizers

19 $$\begin{aligned} s_h(u_h,v_h) = \int _0^T \mathcal J_h(u_h,v_h)\, dt + h^2(\partial _t u_h,\partial _t v_h), \quad s_h^*(z_h,w_h) = (\nabla z_h, \nabla w_h), \end{aligned}$$

defined on the respective semi-discrete spaces

20 $$\begin{aligned} {\mathcal V_h}= H^1(0,T; V_h), \quad {\mathcal W_h}= L^2(0,T; W_h). \end{aligned}$$

We define also the semi-norm and norm

21 $$\begin{aligned} |u_h|_{\mathcal V_h}= s_h(u_h,u_h)^{1/2}, \quad \Vert z_h\Vert _{\mathcal W_h}= s^*_h(z_h,z_h)^{1/2}, \quad u_h \in {\mathcal V_h},\ z_h \in {\mathcal W_h},\ h > 0. \end{aligned}$$

Recall that $W_h = V_h \cap H_0^1(\Omega )$ and whence the Poincaré inequality

22 $$\begin{aligned} \Vert z\Vert _{L^2(\Omega )} \le C \Vert \nabla z\Vert _{L^2(\Omega )}, \quad z \in H_0^{1}(\Omega ), \end{aligned}$$

implies that $\Vert \cdot \Vert _{\mathcal W_h}$ is indeed a norm on ${\mathcal W_h}$.

#### Lemma 3

The semi-norm $|\cdot |_{\mathcal V_h}$ and norm $\Vert \cdot \Vert _{\mathcal W_h}$ satisfy the following inequalities with a constant $C > 0$ that is independent from $h > 0$: lower bounds

23 $$\begin{aligned} G(u_h,z)&\le C |u_h|_{\mathcal V_h}\left( h^{-1} \Vert z\Vert + \Vert z\Vert _{(0,1)}\right) , \quad u_h \in {\mathcal V_h},\ z \in H_0^{(0,1)}, \end{aligned}$$

24 $$\begin{aligned} G(u,z_h)&\le C \Vert z_h\Vert _{{\mathcal W_h}}\left( \Vert u\Vert _{(1,0)} + \Vert u\Vert _{(0,1)}\right) , \quad u \in H^{(1,0)} \cap H^{(0,1)},\ z_h \in {\mathcal W_h}, \end{aligned}$$

upper bounds

25 $$\begin{aligned} |\pi _h u|_{\mathcal V_h}&\le C h (\Vert u\Vert _{(1,1)} + \Vert u\Vert _{(0,2)}), \quad u \in H^{(1,1)} \cap H^{(0,2)}, \end{aligned}$$

26 $$\begin{aligned} \Vert \pi _h z\Vert _{\mathcal W_h}&\le C \Vert z\Vert _{(0,1)}, \quad z \in H^{(0,1)}_0, \end{aligned}$$

and finally, for the semi-norm $|\cdot |_{\mathcal V_h}$ only, the Poincaré type inequality

27 $$\begin{aligned} \Vert u_h\Vert \le C h^{-1} (|u_h|_{\mathcal V_h}+ \Vert u_h\Vert _\omega ), \quad u_h \in {\mathcal V_h}. \end{aligned}$$

#### Proof

We have

$$\begin{aligned} G(u_h,z)= & {} (\partial _t u_h, z) + a(u_h,z) \le \Vert h \partial _t u_h\Vert h^{-1} \Vert z\Vert \\&+ C \int _0^T \mathcal J_h(u_h,u_h) dt \,(h^{-1} \Vert z\Vert + \Vert z\Vert _1), \end{aligned}$$

where the bound for $a(u_h,z)$ follows from (18), analogously to (16), after integration in time. This implies that (23) holds.

The lower bound (24) follows from the Cauchy–Schwarz inequality

$$\begin{aligned} (\partial _t u, z_h) + a(u,z_h) \le \Vert \partial _t u\Vert \Vert z_h\Vert + a(u,u)^{1/2} a(z_h,z_h)^{1/2}, \end{aligned}$$

together with the Poincaré inequality (22).

Towards the upper bound (25), we have

$$\begin{aligned} |\pi _h u|_{\mathcal V_h}^2 = \int _0^T \mathcal J_h(\pi _h u,\pi _h u) + h^2 \Vert \partial _t \pi _h u\Vert ^2 \le C h^2 \Vert u\Vert _{(0,2)}^2 + C h^2 \Vert u\Vert _{(1,1)}^2, \end{aligned}$$

where the bound for the first term follows from (17). The bound for the second term holds by the $H^1$-stability (11) of the interpolator $\pi _h$.

The upper bound (26) follows immediately from the $H^1$-stability of $\pi _h$, and the lower bound (27) follows from (15). $\square$

Observe that, by the lower bound (27),

$$\begin{aligned} \vert \Vert (u_h,z_h)\Vert \vert _h = |u_h|_{\mathcal V_h}+ \Vert u_h\Vert _{\omega } + \Vert z_h\Vert _{{\mathcal W_h}}, \end{aligned}$$

is a norm on ${\mathcal V_h}\times {\mathcal W_h}$.

Let $q \in L^2((0,T) \times \omega )$ and $f \in H^{(0,-1)}$. The Lagrangian

$$\begin{aligned} L_{q,f,h}(u_h,z_h) = \frac{1}{2} \Vert u_h - q\Vert _{\omega }^2 + \frac{1}{2} s_h(u_h,u_h) - \frac{1}{2} s_h^*(z_h,z_h) + G_f(u_h,z_h) \end{aligned}$$

satisfies

$$\begin{aligned} D_{u_h} L_{q,f,h}\, v_h&= (u_h - q, v_h)_{\omega } + s_h(u_h,v_h) + G(v_h,z_h),\\ D_{z_h} L_{q,f,h}\, w_h&= -s^*_h(z_h,w_h) + G(u_h,w_h) - \left\langle f,w_h \right\rangle , \end{aligned}$$

and therefore the critical points $(u_h,z_h) \in {\mathcal V_h}\times {\mathcal W_h}$ of $L_{q,f,h}$ satisfy

28 $$\begin{aligned} A_h[(u_h,z_h),(v_h,w_h)] = (q,v_h)_{\omega } + \left\langle f,w_h \right\rangle , \quad (v_h,w_h) \in {\mathcal V_h}\times {\mathcal W_h}, \end{aligned}$$

where $A_h$ is the symmetric bilinear form

29 $$\begin{aligned}&A_h[(u_h,z_h),(v_h,w_h)] \nonumber \\&\quad = (u_h,v_h)_{\omega } + s_h(u_h,v_h) + G(v_h,z_h) - s^*_h(z_h,w_h) + G(u_h,w_h). \end{aligned}$$

Note that

$$\begin{aligned} A_h[(u_h,z_h),(u_h,-z_h)] = |u_h|_{\mathcal V_h}^2 + \Vert u_h\Vert _{\omega }^2 + \Vert z_h\Vert _{{\mathcal W_h}}^2, \end{aligned}$$

and therefore $A_h$ is weakly coercive in the following sense

30 $$\begin{aligned} \vert \Vert (u_h,z_h)\Vert \vert _h \le C \sup _{(v_h,w_h) \in {\mathcal V_h}\times {\mathcal W_h}} \frac{A_h[(u_h,z_h),(v_h,w_h)]}{\vert \Vert (v_h,w_h)\Vert \vert _h}, \quad (u_h,z_h) \in {\mathcal V_h}\times {\mathcal W_h}. \end{aligned}$$

The Babuska-Lax-Milgram theorem implies that the equation (28) has a unique solution in ${\mathcal V_h}\times {\mathcal W_h}$.

### Error estimates

In this section we show that the solution $(u_h, z_h)$ of (28) satisfies $u_h \rightarrow u$ in $(T_1, T_2) \times B$ as $h \rightarrow 0$, with the convergence rate (7). Here *u* is a smooth enough solution of the unstable data assimilation problem in the continuum. We use the shorthand notation

$$\begin{aligned} \Vert u\Vert _{*} = \Vert u\Vert _{(1,1)} + \Vert u\Vert _{(0,2)}. \end{aligned}$$

#### Lemma 4

Let $u \in H^{(1,1)} \cap H^{(0,2)}$ and define $f = \partial _t u - \Delta u$ and $q = u|_{(0,T) \times \omega }$. Let $(u_h, z_h) \in {\mathcal V_h}\times {\mathcal W_h}$ be the solution of (28). Then there exists $C > 0$ such that for all $h \in (0,1)$,

$$\begin{aligned} \vert \Vert (u_h - \pi _h u,z_h)\Vert \vert _h&\le C h \Vert u\Vert _{*}. \end{aligned}$$

#### Proof

The equations $\partial _t u - \Delta u = f$ and $u|_\omega = q$ are equivalent with

31 $$\begin{aligned} G(u,w)&= \left\langle f,w \right\rangle , \quad w \in L^2(0,T; H^1_0(\Omega )), \nonumber \\ (q-u,v)_{\omega }&= 0, \quad v \in L^2((0,T) \times \omega ), \end{aligned}$$

and the Eqs. (28) and (31) imply for all $v_h \in {\mathcal V_h}$ and $w_h \in {\mathcal W_h}$ that

32 $$\begin{aligned}&A_h[(u_h-\pi _h u, z_h),(v_h,w_h)] = (u-\pi _h u,v_h)_{\omega } + G(u-\pi _h u, w_h) - s_h(\pi _h u,v_h). \end{aligned}$$

By (30) it is enough to show that

$$\begin{aligned} A_h[(u_h - \pi _h u, z_h),(v_h,w_h)] \le C h \Vert u\Vert _{*} \vert \Vert (v_h,w_h)\Vert \vert _h. \end{aligned}$$

We use (12) to bound the first term in (32),

$$\begin{aligned} (u - \pi _h u,v_h)_{\omega } \le \Vert u - \pi _h u\Vert _{\omega } \Vert v_h\Vert _{\omega } \le C h \Vert u\Vert _{(0,1)} \Vert v_h\Vert _{\omega }, \end{aligned}$$

for the second term we use (24) and (12),

$$\begin{aligned}&G(u - \pi _h u, w_h) \le C \left( \Vert u - \pi _h u\Vert _{(1,0)}\right. \\&\quad \left. + \Vert u - \pi _h u\Vert _{(0,1)}\right) \Vert w_h\Vert _{{\mathcal W_h}} \le C h \Vert u\Vert _{*} \Vert w_h\Vert _{{\mathcal W_h}}, \end{aligned}$$

and for the third term we use (25),

$$\begin{aligned} s_h(\pi _h u, v_h) \le |\pi _h u|_{\mathcal V_h}|v_h|_{\mathcal V_h}\le C h \Vert u\Vert _{*} |v_h|_{\mathcal V_h}. \end{aligned}$$

$$\square $$

#### Theorem 3

Let $\omega , B \subset \Omega$, $0< T_1< T_2 < T$ and $\kappa \in (0,1)$ be as in Theorem 1. Let $u \in H^{(1,1)} \cap H^{(0,2)}$ and define $f = \partial _t u - \Delta u$ and $q = u|_{(0,T) \times \omega }$. Let $(u_h, z_h) \in {\mathcal V_h}\times {\mathcal W_h}$ be the solution of (28). Then there exists $C > 0$ such that for all $h \in (0,1)$

$$\begin{aligned} \Vert u_h - u\Vert _{L^2(T_1, T_2; H^1(B))}&\le C h^\kappa (\Vert u\Vert _{*} + \Vert f\Vert ). \end{aligned}$$

#### Proof

We define the functional $\left\langle r,w \right\rangle = G(u_h - u, w)$, $w \in H_0^{(0,1)}$. That is, *r* is the residual $r = (\partial _t - \Delta )(u_h - u)$ in the weak sense. The equation $\partial _t u - \Delta u = f$ implies that

$$\begin{aligned} \left\langle r,w \right\rangle = G(u_h, w) - \left\langle f, w \right\rangle . \end{aligned}$$

By taking $v_h=0$ in (28) we get $G(u_h, w_h) = \left\langle f,w_h \right\rangle + s^*_h(z_h,w_h)$, $w_h \in {\mathcal W_h}$, and therefore

33 $$\begin{aligned} \left\langle r,w \right\rangle&= G(u_h,w) - \left\langle f,w \right\rangle - G(u_h, \pi _h w) + G(u_h, \pi _h w)\nonumber \\&= G(u_h,w - \pi _h w) - \left\langle f,w-\pi _h w \right\rangle + s^*_h(z_h,\pi _h w), \quad w \in H_0^{(0,1)}. \end{aligned}$$

We arrive to the estimate

34 $$\begin{aligned} \Vert r\Vert _{(0,-1)} \le C (|u_h|_{{\mathcal V_h}} + \Vert z_h\Vert _{{\mathcal W_h}} + h \Vert f\Vert ) \end{aligned}$$

after we bound the first term in (33) by using (23) and (11)–(12)

35 $$\begin{aligned} G(u_h, w - \pi _h w)&\le C |u_h|_{\mathcal V_h}\left( h^{-1} \Vert w - \pi _h w\Vert + \Vert w - \pi _h w\Vert _{(0,1)}\right) \nonumber \\&\le C |u_h|_{\mathcal V_h}\Vert w\Vert _{(0,1)}, \end{aligned}$$

the second term by using (12)

$$\begin{aligned} (f, w - \pi _h w)&\le \Vert f\Vert \Vert w - \pi _h w\Vert \le C h \Vert f\Vert \Vert w\Vert _{(0,1)}, \end{aligned}$$

and the last term by using (26)

$$\begin{aligned} s^*_h(z_h, \pi _h w) \le \Vert z_h\Vert _{\mathcal W_h}\Vert \pi _h w\Vert _{\mathcal W_h}\le C \Vert z_h\Vert _{\mathcal W_h}\Vert w\Vert _{(0,1)}. \end{aligned}$$

We bound the first term in (34) by using Lemma 4 and (25)

$$\begin{aligned} |u_h|_{\mathcal V_h}\le |u_h - \pi _h u|_{\mathcal V_h}+ |\pi _h u|_{\mathcal V_h}\le C h \Vert u\Vert _{*}, \end{aligned}$$

and observe that the analogous bound for the second term follows immediately from Lemma 4. Thus

$$\begin{aligned} \Vert r\Vert _{(0,-1)} \le C h(\Vert u\Vert _{*} + \Vert f\Vert ). \end{aligned}$$

By applying Theorem 1 to $u_h - u$, and using Lemma 4 and (12) to bound the term

$$\begin{aligned} \Vert u_h - u\Vert _\omega \le \Vert u_h - \pi _h u\Vert _\omega + \Vert \pi _h u - u\Vert _\omega \le C h \Vert u\Vert _{*} + C h \Vert u\Vert _{(0,1)} \end{aligned}$$

we see that

$$\begin{aligned}&\Vert u_h - u\Vert _{L^2(T_1,T_2; H^1(B))} \le C h^\kappa (\Vert u\Vert _{*} + \Vert f\Vert )^\kappa (\Vert u_h - u\Vert + h(\Vert u\Vert _{*} + \Vert f\Vert ))^{1-\kappa }. \end{aligned}$$

It remains to show the bound

36 $$\begin{aligned} \Vert u_h-u\Vert \le C \Vert u\Vert _{*}. \end{aligned}$$

The Poincaré type inequality (27) implies

$$\begin{aligned} \Vert u_h - \pi _h u\Vert \le C h^{-1} |u_h - \pi _h u|_{{\mathcal V_h}} + C h^{-1} \Vert u_h - \pi _h u\Vert _\omega \le C \Vert u\Vert _{*}, \end{aligned}$$

where we used again Lemma 4. The inequality (36) follows since $\Vert \pi _h u - u\Vert$ can be bounded by $\Vert u\Vert _{*}$, in fact, even by $C h^2 \Vert u\Vert _{*}$. $\square$

### The effect of perturbations in data

In practice the data *f*, *q* in (3) are typically polluted by measurement errors. Such effects can easily be included in the analysis and we show in this section how the above results must be modified to account for this case. We consider the case where *f*, *q* in (28) are replaced by the perturbed counterparts

37 $$\begin{aligned} \tilde{q} = u|_{(0, T) \times \omega } + \delta q, \quad \tilde{f} = f + \delta f, \end{aligned}$$

where *u* is the solution to the underlying unpolluted problem, that is, $\partial _t u - \Delta u = f$. We assume that the perturbations $\delta q, \delta f$ satisfy $\delta f \in H^{-1}(\Omega )$ and $\delta q \in L^2(\omega )$, and use the following measure of the size of the perturbation

$$\begin{aligned} \delta (\tilde{q},\tilde{f}) = \Vert \delta q\Vert _{L^2(0,T;L^2(\omega ))}+\Vert \delta f\Vert _{(0,-1)}. \end{aligned}$$

The critical points $(u_h,z_h) \in \mathcal {V}_h \times \mathcal {W}_h$ of the perturbed Lagrangian $L_{\tilde{q}, \tilde{f},h}$ satisfy

38 $$\begin{aligned} A_h[(u_h,z_h),(v_h,w_h)] = (\tilde{q},v_h)_\omega + \langle \tilde{f},w_h \rangle , \quad (v_h,w_h) \in {\mathcal V_h}\times {\mathcal W_h}. \end{aligned}$$

We consider a perturbation of *q* in $L^2$ since this is used in the data fitting term in the Lagrangian. It should be noted that the formulation (38) remains well defined for rougher perturbations $\delta q$, but they will lead to worse estimates in *h*.

First we consider again the residual quantities as in Lemma 4.

#### Lemma 5

Let $u \in H^{(1,1)} \cap H^{(0,2)}$ and define $\tilde{f}$ and $\tilde{q}$ by (37). Let $(u_h, z_h) \in {\mathcal V_h}\times {\mathcal W_h}$ be the solution of (38). Then there exists $C > 0$ such that for all $h \in (0,1)$

$$\begin{aligned} \vert \Vert (u_h - \pi _h u,z_h)\Vert \vert _h&\le C( h \Vert u\Vert _{*} + \delta (\tilde{q},\tilde{f})). \end{aligned}$$

#### Proof

Proceeding as in the proof of Lemma 4, the Eqs. (38) and (31) imply for all $v_h \in {\mathcal V_h}$ and $w_h \in {\mathcal W_h}$ that

39 $$\begin{aligned}&A_h[(u_h-\pi _h u, z_h),(v_h,w_h)] = (u-\pi _h u,v_h)_{\omega } +(\delta q, v_h)_\omega \nonumber \\&\quad + G(u-\pi _h u, w_h)+\left\langle \delta f,w_h \right\rangle - s_h(\pi _h u,v_h). \end{aligned}$$

The only terms that are not covered by the previous analysis are those with the perturbations, and we have

$$\begin{aligned} (\delta q,v_h)_\omega + \left\langle \delta f,w_h \right\rangle \le \delta (\tilde{q},\tilde{f}) \vert \Vert (v_h,w_h)\Vert \vert _h \end{aligned}$$

The choice (19) of the dual stabilizer $s^*$ guarantees that $\Vert w\Vert _{(0,1)} \le \Vert w\Vert _{\mathcal W_h}$. $\square$

The error estimate of Theorem 3 may now be modified to include the perturbations.

#### Theorem 4

Let $\omega , B \subset \Omega$, $0< T_1< T_2 < T$ and $\kappa \in (0,1)$ be as in Theorem 1. Let $u \in H^{(1,1)} \cap H^{(0,2)}$ and define $\tilde{f}$ and $\tilde{q}$ by (37). Let $(u_h, z_h) \in {\mathcal V_h}\times {\mathcal W_h}$ be the solution of (38). Then there exists $C > 0$ such that for all $h \in (0,1)$

$$\begin{aligned}&\Vert u_h - u\Vert _{L^2(T_1, T_2; H^1(B))}\\&\quad \le C (h(\Vert u\Vert _{*} + \Vert f\Vert )+ \delta (\tilde{q},\tilde{f}))^\kappa (\Vert u\Vert _{*} + h \Vert f\Vert + h^{-1} \delta (\tilde{q},\tilde{f}))^{(1-\kappa )}. \end{aligned}$$

#### Proof

We proceed as in the proof of Theorem 3, but due to the perturbation, the Galerkin orthogonality (33) no longer holds exactly and we obtain

$$\begin{aligned} \left\langle r,w \right\rangle&= G(u_h,w - \pi _h w) - \left\langle f,w-\pi _h w \right\rangle + \left\langle \delta f,\pi _h w \right\rangle + s^*_h(z_h,\pi _h w), \end{aligned}$$

for $w \in H_0^{(0,1)}$. Analogously to the proof of Theorem 3, we obtain

40 $$\begin{aligned} \Vert r\Vert _{(0,-1)} \le C (|u_h|_{{\mathcal V_h}} + \Vert z_h\Vert _{{\mathcal W_h}} + h \Vert f\Vert + \Vert \delta f\Vert _{(0,-1)}). \end{aligned}$$

We bound the first term in (40) by using Lemma 5 and (25)

$$\begin{aligned} |u_h|_{\mathcal V_h}\le |u_h - \pi _h u|_{\mathcal V_h}+ |\pi _h u|_{\mathcal V_h}\le C( h \Vert u\Vert _{*} + \delta (\tilde{q},\tilde{f})), \end{aligned}$$

and the second term by using again Lemma 5. Thus

$$\begin{aligned} \Vert r\Vert _{(0,-1)} \le C h(\Vert u\Vert _{*} + \Vert f\Vert )+ C\delta (\tilde{q},\tilde{f}) . \end{aligned}$$

As before, applying Theorem 1 to $u_h - u$, and using Lemma 5 and (12) to bound the term $\Vert u_h - u\Vert _\omega$ leads to

$$\begin{aligned}&\Vert u_h - u\Vert _{L^2(T_1,T_2; H^1(B)}\\&\quad \le C (h (\Vert u\Vert _{*} + \Vert f\Vert ) + \delta (\tilde{q},\tilde{f}))^\kappa (\Vert u_h - u\Vert + h(\Vert u\Vert _{*} + \Vert f\Vert ) + \delta (\tilde{q},\tilde{f}))^{1-\kappa }. \end{aligned}$$

It remains to bound $\Vert u_h - u\Vert$. The Poincaré type inequality (27) implies

$$\begin{aligned} \Vert u_h - \pi _h u\Vert&\le C h^{-1} |u_h - \pi _h u|_{{\mathcal V_h}} + C h^{-1} \Vert u_h - \pi _h u\Vert _\omega \\&\le C (\Vert u\Vert _{*} + h^{-1} \delta (\tilde{q},\tilde{f})), \end{aligned}$$

where we used again Lemma 5. $\square$

According to Theorem 4, the error of the numerical approximation can diverge if no function $\tilde{u}$ exists such that $\partial _t \tilde{u} - \Delta \tilde{u} = \tilde{f}$ and $\tilde{u}\vert _{(0,T) \times \omega } = \tilde{q}$. On the other hand, in the context of a specific application, an estimate on the noise level might be available, and this can be used to choose a suitable mesh size. That is, assuming that there exists $h_0>0$ such that

$$\begin{aligned} \delta (\tilde{q},\tilde{f}) \le h_0 (\Vert u\Vert _{*} + \Vert f\Vert ), \end{aligned}$$

then for $h>h_0$ there holds

$$\begin{aligned} \Vert u_h - u\Vert _{L^2(T_1, T_2; H^1(B))}&\le C h^\kappa (\Vert u\Vert _{*} + \Vert f\Vert )^\kappa . \end{aligned}$$

## A framework for stabilization

Before proceeding to the stable problem, we introduce an abstract stabilization framework based on the essential features of the model case in Sect. 3.1.

Let $s_h$ and $s_h^*$ be bilinear forms on the spaces ${\mathcal V_h}$ and ${\mathcal W_h}$, respectively. Let $|\cdot |_{\mathcal V_h}$ be a semi-norm on ${\mathcal V_h}$ and let $\Vert \cdot \Vert _{\mathcal W_h}$ be a norm on ${\mathcal W_h}$. We relax (21) by requiring only that $s_h$ and $s_h^*$ are continuous with respect to $|\cdot |_{\mathcal V_h}$ and $\Vert \cdot \Vert _{\mathcal W_h}$, that is,

41 $$\begin{aligned} s_h(u_h,u_h) \le C |u_h|_{\mathcal V_h}^2, \quad s^*_h(z_h,z_h) \le C \Vert z_h\Vert _{\mathcal W_h}^2, \quad u_h \in {\mathcal V_h},\ z_h \in {\mathcal W_h},\ h > 0. \end{aligned}$$

Let $\Vert \cdot \Vert _*$ be the norm of a continuously embedded subspace $H^*$ of the energy space (2). The space $H^*$ encodes the apriori smoothness. We relax the lower bounds (23) and (24) as follows

42 $$\begin{aligned} G(u_h,z - \pi _h z)&\le C |u_h|_{\mathcal V_h}\Vert z\Vert _{(0,1)},&u_h \in {\mathcal V_h},\ z \in H_0^{(0,1)}, \end{aligned}$$

43 $$\begin{aligned} G(u - \pi _h u,z_h)&\le C h \Vert z_h\Vert _{{\mathcal W_h}} \Vert u\Vert _{*},&u \in H^{*},\ z_h \in {\mathcal W_h}, \end{aligned}$$

where $\pi _h$ is an interpolator satisfying (11)–(13). We replace the upper bound (25) by its abstract analogue

44 $$\begin{aligned} |\pi _h u|_{\mathcal V_h}&\le C h \Vert u\Vert _{*},\quad&u \in H^{*}, \end{aligned}$$

and require that (26) and (27) hold as before. Finally, in the abstract setting, we assume that the weak coercivity (30) holds where $A_h$ and $\vert \Vert \cdot \Vert \vert _h$ are defined as above.

It can be verified that the following analogue of Theorem 3 holds under these assumptions.

### Theorem 5

Let $\omega , B \subset \Omega$, $0< T_1< T_2 < T$ and $\kappa \in (0,1)$ be as in Theorem 1. Let $u \in H^{*}$ and define $f = \partial _t u - \Delta u$ and $q = u|_\omega$. Suppose that the primal and dual stabilizers satisfy (41)–(44), (26), (27) and (30). Then (28) has a unique solution $(u_h, z_h) \in {\mathcal V_h}\times {\mathcal W_h}$, and there exists $C > 0$ such that for all $h \in (0,1)$

$$\begin{aligned} \Vert u_h - u\Vert _{L^2(T_1, T_2; H^1(B))}&\le C h^\kappa (\Vert u\Vert _{*} + \Vert f\Vert ). \end{aligned}$$

## The stable problem

Let us now consider the stable problem with the additional lateral boundary condition $u|_{(0,T) \times \partial \Omega } = 0$. We may impose the boundary condition on the discrete level by changing the space ${\mathcal V_h}$, defined previously by (20), to

$$\begin{aligned} {\mathcal V_h}= H^1(0,T; W_h). \end{aligned}$$

In the stable case we do not need an inequality analogous to (36) since the estimate in Theorem 2 does not contain an apriori term, that is, a term analogous to $\Vert u\Vert _{L^2((0,T) \times \Omega )}$ in Theorem 1. In the previous section, the Poincaré type inequality (27) was used only to obtain (36), and whence we can relax the conditions there by dropping (27). However, we still need to require that $\vert \Vert \cdot \Vert \vert _h$ is a norm on ${\mathcal V_h}\times {\mathcal W_h}$.

We will now proceed to a concrete case. The choices in Sect. 4 work also in the stable case, however, the additional structure allows us to choose a weaker stabilization. The use of a weaker stabilization is motivated by the fact that it leads to a weaker coupling of the two heat equations in (28), which again can be exploited when solving (28) in practice. See [10] for a computational implementation based on this.

The stabilizers and semi-norms are chosen as follows,

45 $$\begin{aligned}&s_h(u_h,u_h) = \Vert h \nabla u_h(0, \cdot )\Vert _{L^2(\Omega )}^2, \quad s_h^*(z_h,z_h) = (\nabla z_h,\nabla z_h), \end{aligned}$$

46 $$\begin{aligned}&|u_h|_{\mathcal V_h}= s_h(u_h,u_h)^{1/2} + \Vert h \partial _t u_h\Vert , \quad \Vert z_h\Vert _{\mathcal W_h}= s^*_h(z_h,z_h)^{1/2}, \end{aligned}$$

and we define $H^* = H_0^{(1,1)}$. To counter the lack of primal stabilization on most of the cylinder $(0,T) \times \Omega$, we choose $\pi _h$ to be the orthogonal projection $\pi _h : H_0^1(\Omega ) \rightarrow W_h$ with respect to the inner product $(\nabla u, \nabla v)_{L^2(\Omega )}$. That is, $\pi _h$ is defined by

47 $$\begin{aligned} (\nabla \pi _h u, \nabla v_h)_{L^2(\Omega )} = (\nabla u, \nabla v_h)_{L^2(\Omega )}, \quad u \in H_0^1(\Omega ),\ v_h \in W_h. \end{aligned}$$

As $\Omega$ is a convex polyhedron, this choice satisfies the estimates (11)–(12), see e.g. [16, Th. 3.12-18].

### Lemma 6

The choices (45)–(47) satisfy the properties (41)–(44), (26) and (30). Moreover, $\vert \Vert \cdot \Vert \vert _h$ is a norm on ${\mathcal V_h}\times {\mathcal W_h}$.

### Proof

It is clear that the continuities (41) hold. We begin with the lower bound (42). By the orthogonality (47),

$$\begin{aligned} G(u_h, z- \pi _h z) = (\partial _t u_h, z- \pi _h z) \le \Vert h \partial _t u_h\Vert h^{-1} \Vert z- \pi _h z\Vert \le C \Vert h \partial _t u_h\Vert \Vert z\Vert _{(0,1)}. \end{aligned}$$

Towards the lower bound (43), we use the orthogonality (47) as above,

$$\begin{aligned} G(u - \pi _h u, z_h) = (\partial _t u - \pi _h \partial _t u, z_h) \le C h \Vert u\Vert _{(1,1)} \Vert z_h\Vert . \end{aligned}$$

The bound (43) follows from the Poincaré inequality (22).

The bound (44) follows from the continuity of the trace

48 $$\begin{aligned} \Vert \nabla u(0, \cdot )\Vert _{L^2(\Omega )} \le \Vert u\Vert _{(1,1)}, \end{aligned}$$

and the continuity of the projection $\pi _h$. The bound (26) follows immediately from the continuity of $\pi _h$.

We turn to the weak coercivity (30). The essential difference between the unstable and the stable case is that in the latter case $\partial _t u_h \in {\mathcal W_h}$ when $u_h \in {\mathcal V_h}$. We have

$$\begin{aligned} A_h[(u_h,z_h),(0,\partial _t u_h)]= & {} - s^*_h(z_h,\partial _t u_h) + G(u_h,\partial _t u_h) \\= & {} \Vert \partial _t u_h\Vert ^2 + a(u_h, \partial _t u_h) - a(z_h, \partial _t u_h), \end{aligned}$$

and thus using bilinearity of $A_h$,

49 $$\begin{aligned} A_h[(u_h,z_h),(u_h,\alpha h^2 \partial _t u_h - z_h)]&= s_h(u_h,u_h) + \alpha \Vert h \partial _t u_h\Vert ^2 + \Vert u_h\Vert _\omega ^2 + s^*_h(z_h,z_h) \nonumber \\&\quad + \alpha h^2 a(u_h, \partial _t u_h) - \alpha h^2 a(z_h, \partial _t u_h), \end{aligned}$$

where $\alpha > 0$. We will establish (30) by showing that there is $\alpha \in (0,1)$ such that

50 $$\begin{aligned} \vert \Vert (u_h, w_h - z_h)\Vert \vert _h&\le C\vert \Vert (u_h,z_h)\Vert \vert _h, \end{aligned}$$

51 $$\begin{aligned} \vert \Vert (u_h,z_h)\Vert \vert _h^2&\le C A_h[(u_h,z_h),(u_h,w_h-z_h)], \end{aligned}$$

where $$w_h = \alpha h^2 \partial _t u_h$$.

Towards (50) we observe that

$$\begin{aligned} \vert \Vert (u_h,w_h-z_h)\Vert \vert _h^2&= \vert \Vert (u_h,z_h)\Vert \vert _h^2 - 2 s^*_h(z_h,w_h) + s^*_h(w_h,w_h)\\&\le 2 \vert \Vert (u_h,z_h)\Vert \vert _h^2 + 2 s^*_h(w_h,w_h). \end{aligned}$$

We use the discrete inverse inequality (10) to bound the second term

$$\begin{aligned} s^*_h(w_h,w_h) = \alpha ^2 h^4 \Vert \partial _t \nabla u_h\Vert ^2 \le C \alpha ^2 h^2 \Vert \partial _t u_h\Vert ^2 \le C \alpha ^2 \vert \Vert (u_h,z_h)\Vert \vert ^2, \quad \alpha > 0. \end{aligned}$$

It remains to show (51). Towards bounding the first cross term in (49) we observe that

$$\begin{aligned} 2 a(u_h, \partial _t u_h)&= \int _0^T \partial _t \Vert \nabla u_h(t, \cdot )\Vert _{L^2(\Omega )}^2 dt = \Vert \nabla u_h(T, \cdot )\Vert _{L^2(\Omega )}^2 - \Vert \nabla u_h(0, \cdot )\Vert _{L^2(\Omega )}^2. \end{aligned}$$

Hence $\alpha h^2 a(u_h, \partial _t u_h) \ge -\alpha s_h(u_h,u_h) / 2.$ We use the arithmetic-geometric inequality,

$$\begin{aligned} ab \le (4\epsilon )^{-1} a^2 + \epsilon b^2, \quad a,b \in \mathbb R,\ \epsilon > 0, \end{aligned}$$

and the discrete inverse inequality (10) to bound the second cross term in (49),

$$\begin{aligned} \alpha h^2 a(z_h, \partial _t u_h)\le & {} \alpha (4\epsilon )^{-1} a(z_h,z_h) + \alpha \epsilon h^4 \Vert \partial _t \nabla u_h\Vert ^2\\\le & {} \alpha (4\epsilon )^{-1} a(z_h,z_h) + C \alpha \epsilon \Vert h \partial _t u_h\Vert ^2. \end{aligned}$$

Choosing $\epsilon = 1 / (2 C)$ we obtain

$$\begin{aligned}&A_h[(u_h,z_h),(u_h,w_h - z_h)]\\&\quad \ge (1-\alpha /2) s_h(u_h,u_h) + \alpha \Vert h \partial _t u_h\Vert ^2 / 2 + \Vert u_h\Vert _\omega ^2 + (1-C \alpha /2) s^*_h(z_h,z_h), \end{aligned}$$

and therefore (51) holds with small enough $\alpha > 0$.

Finally, using the Poincaré inequality (22), we see that $\vert \Vert (u_h,z_h)\Vert \vert _h = 0$ implies that $z_h=0$ and $u_h(0, \cdot ) = 0$. As also $\partial _t u_h = 0$, we have $u_h=0$, and therefore $\vert \Vert \cdot \Vert \vert _h$ is a norm. $\square$

In the stable case we have the following convergence rate.

### Theorem 6

Let $\omega \subset \Omega$ be open and non-empty and let $0< T_1 < T$. Suppose that (A2) holds. Let $u \in H^{*}$ and define $f = \partial _t u - \Delta u$ and $q = u|_\omega$. Suppose that the primal and dual stabilizers satisfy (41)–(44), (26) and (30). Then (28) has a unique solution $(u_h, z_h) \in {\mathcal V_h}\times {\mathcal W_h}$, and there exists $C > 0$ such that for all $h \in (0,1)$

$$\begin{aligned}&\Vert u_h - u\Vert _{C(T_1, T; L^2(\Omega ))} + \Vert u_h - u\Vert _{L^2(T_1, T; H^1(\Omega ))} + \Vert u_h - u\Vert _{H^1(T_1, T; H^{-1}(\Omega ))}\\&\quad \le C h (\Vert u\Vert _{*} + \Vert f\Vert ). \end{aligned}$$

The proof is analogous to that of Theorem 3, however, we give it here for the sake of completeness.

### Proof

We begin again by showing the estimate

52 $$\begin{aligned} \vert \Vert (u_h - \pi _h u,z_h)\Vert \vert _h&\le C h \Vert u\Vert _{*}. \end{aligned}$$

The weak coercivity (30) implies that in order to show (52) it is enough bound the three terms on the right hand side of (32). For the first of them, that is, $(u - \pi _h u,v_h)_{\omega }$, we use (12) as in the proof of Lemma 4. The lower bound (43) applies to the second term $G(u - \pi _h u, w_h)$, and for the third one we use the continuity (41) and the upper bound (44),

$$\begin{aligned} s_h(\pi _h u, v_h) \le C |\pi _h u|_{\mathcal V_h}|v_h|_{\mathcal V_h}\le C h \Vert u\Vert _{*} |v_h|_{\mathcal V_h}. \end{aligned}$$

We define the residual *r* as in the proof of Theorem 3, and show next that (34) holds. It is enough to bound the three terms on the right hand side of (33). The lower bound (42) applies to the first term $G(u_h, w - \pi _h w)$, for the second term $(f, w - \pi _h w)$ we use (12) as in the proof of Theorem 3, and for the third term we use the continuity (41) and the upper bound (26)

$$\begin{aligned} s^*_h(z_h, \pi _h w) \le C \Vert z_h\Vert _{\mathcal W_h}\Vert \pi _h w\Vert _{\mathcal W_h}\le C \Vert z_h\Vert _{\mathcal W_h}\Vert w\Vert _{(0,1)}. \end{aligned}$$

The inequalities (34), (52) and (44) imply

$$\begin{aligned} \Vert r\Vert _{(0,-1)} \le C (|u_h - \pi _h u|_{{\mathcal V_h}} + |\pi _h u|_{\mathcal V_h}+ \Vert z_h\Vert _{{\mathcal W_h}} + h \Vert f\Vert ) \le C h (\Vert u\Vert _* + \Vert f\Vert ). \end{aligned}$$

Finally, Theorem 2 implies that

$$\begin{aligned}&\Vert u_h - u\Vert _{C(T_1, T; L^2(\Omega ))} + \Vert u_h - u\Vert _{L^2(T_1, T; H^1(\Omega ))} + \Vert u_h - u\Vert _{H^1(T_1, T; H^{-1}(\Omega ))}\\&\quad \le C \Vert u_h - u\Vert _\omega + C h (\Vert u\Vert _{*} + \Vert f\Vert ). \end{aligned}$$

The claim follows by using (52) and (12),

$$\begin{aligned} \Vert u_h - u\Vert _\omega \le \Vert u_h - \pi _h u\Vert _\omega + \Vert \pi _h u - u\Vert _\omega \le C h \Vert u\Vert _*. \end{aligned}$$

Here we used also the assumption that $H^*$ is a continuously embedded subspace of the energy space (2), namely, this implies that the embedding $H^* \subset H^{(0,1)}$ is continuous. $\square$

### Remark 1

If the data *q*, *f* is perturbed in the stable case, the data assimilation problem behaves like a typical well posed problem, that is, the term $\delta (\tilde{q}, \tilde{f})$ needs to be added on the right-hand side of the estimate in Theorem 6, but this time without any negative power of *h*.

## Conclusion

In the present paper our aim was to show how methods known from the theory of stabilized finite element methods can be applied to the design of computational methods for non-stationary data assimilation problems by first discretizing and then regularizing the corresponding 4DVAR optimization system.

A key feature of this framework is that the error analysis is based on numerical stability of residual quantities that are independent of the stability properties of the continuous model. The residual quantities are then bounded by using conditional stability estimates, based on Carleman estimates, to derive error estimates that reflect the approximation properties of the finite element space and the stability of the continuous problem in an optimal way. An upshot of this approach is that it gives a clear lead on how to design regularization for a given problem in order to obtain the best accuracy of the approximation with the least effect of perturbations in data.

Observe that the stabilization operators proposed herein are not unique, for instance it is straightforward to show that the dual stabilizer in Eq. (19) may be chosen as the first part of the primal stabilizer, leading to similar error estimates for unperturbed data. The error estimates also gives an indication on what form Carleman estimates should take to make them immediately applicable for error analysis.

## Appendix: Continuum estimates

Theorem 1 is a consequence of the so-called three cylinders inequality that goes back to [19], see [27, 28] for an overview of the related literature. The variant of the inequality, needed in the above convergence analysis, seems not to appear in the literature, and we prove it here by using the Carleman estimate [20].

Theorem 2 is a variant of [14], the difference being that in [14] the domain $\Omega$ is assumed to have $C^2$-smooth boundary. We outline below the modifications needed in the case that $\Omega$ is a convex polyhedron.

We use the notation $B(x,r) = \{y \in \Omega ;\ d(y,x) < r\}$, $x \in \Omega$, $r>0$, where *d* is the Euclidean distance.

### Theorem 7

(Three cylinders inequality) Let $x_0 \in \Omega$ and $0< r_1< r_2 < d(x_0, \partial \Omega )$. Write $B_j = B(x_0, r_j)$, $j=1,2$. Let $T > 0$ and $0< \epsilon < T / 2$. Then there are $C > 0$ and $\kappa \in (0,1)$ such that for all $u \in C^2(\mathbb R\times \Omega )$

$$\begin{aligned} \Vert u\Vert _{L^2(\epsilon , T - \epsilon ; H^1(B_2))} \le C (\Vert u\Vert _{L^2(0, T; H^1(B_1))} + \Vert L u\Vert _{L^2((0, T) \times \Omega )})^\kappa \Vert u\Vert _{L^2(0, T;H^1(\Omega ))}^{1-\kappa }. \end{aligned}$$

### Proof

Let $0< r_0 < r_1$ and $r_2< r_3< r_4 < d(x_0, \partial \Omega )$. Define $B_j = B(x_0, r_j)$, $j=0,3,4$. We choose non-positive $\rho _1 \in C^\infty (\Omega )$ such that $\rho _1 > - r_0$ in $B_0$ and that $\rho _1(x) = -d(x,x_0)$ outside $B_0$. Define $I_1 = (\epsilon ,T-\epsilon )$ and $I_2 = (\epsilon /2,T-\epsilon /2)$, and choose non-positive $\rho _2 \in C^\infty (\mathbb R)$ such that $\rho _2 \le -r_3$ outside $I_2$ and $\rho _2 = 0$ in $I_1$. We define $\rho (t,x) = \rho _1(x) + \rho _2(t)$ and $\phi = e^{\alpha \rho }$ where $\alpha > 0$.

We will apply [20, Th. 1.1] to the heat operator $L = \partial _t - \Delta$, with the above weight function $\phi$. Condition (1.5) in the theorem reduces to

53 $$\begin{aligned} D^2 \phi (\zeta , \overline{\zeta })> 0, \quad \zeta = \xi + i \tau \nabla \phi ,\ |\xi | = \tau |\nabla \phi |,\ \tau > 0,\ \xi \in \mathbb R^n, \end{aligned}$$

where $D^2 \phi$ is the Hessian of $\phi$ with respect to *x*. We have $\nabla \phi = \alpha \phi \nabla \rho$ and

$$\begin{aligned} D^2 \phi (\zeta , \overline{\zeta })&= \alpha \phi (\alpha |\nabla \rho \cdot \zeta |^2 + D^2 \rho (\zeta , \overline{\zeta }))\\&\ge \alpha \phi ( \alpha (\tau \alpha \phi |\nabla \rho |^2)^2 - \mu (\tau \alpha \phi |\nabla \rho |)^2), \end{aligned}$$

where $\mu$ is a constant depending only on $\rho$. As $|\nabla \rho | = 1$ outside $B_0$, condition (53) holds in $\overline{B_4} \setminus B_0$ for large enough $\alpha > 0$. Let $\chi \in C_0^\infty ((0,T) \times (B_4 \setminus B_0))$ satisfy $\chi = 1$ in $I_2 \times (B_3 \setminus B_1)$, and set $w = \chi u$. Then [20, Th. 1.1] implies that for large $\tau > 0$,

54 $$\begin{aligned}&\int _0^T \int _{B_4 \setminus B_0} ( \tau |\nabla w|^2 + \tau ^3 |w|^2) e^{2 \tau \phi }\, dx dt \le C \int _0^T \int _{B_4 \setminus B_0} |L w|^2 e^{2 \tau \phi }\, dx dt. \end{aligned}$$

We define $\Phi (r) = e^{-\alpha r}$, $I = (0,T)$ and

$$\begin{aligned} Q_1 = I_2 \times B_1, \quad Q_2 = ((I \setminus I_2) \times B_4) \cup (I \times (B_4 \setminus B_3)). \end{aligned}$$

Observe that $\phi \le \Phi (r_3)$ in $Q_2$, and that the commutator $[L, \chi ]$ vanishes outside $Q_1 \cup Q_2$. Hence the right-hand side of (54) is bounded by a constant times

55 $$\begin{aligned}&\int _{(0,T) \times B_4} |L u|^2 e^{2 \tau \phi } dx dt + \int _{Q_1 \cup Q_2} |[L, \chi ]u|^2 e^{2 \tau \phi } dx dt \nonumber \\&\quad \le e^{2 \tau } \Vert Lu\Vert _{L^2((0,T) \times B_4)}^2 + e^{2 \tau } \Vert u\Vert _{L^2(0,T; H^1(B_1))}^2 + e^{2\tau \Phi (r_3)} \Vert u\Vert _{L^2(0,T; H^1(B_4))}^2. \end{aligned}$$

The left-hand side of (54) is bounded from below by

56 $$\begin{aligned}&\int _{I_1 \times (B_2 \setminus B_1)} \left( \tau |\nabla u|^2 + \tau ^3 |u|^2 \right) e^{2 \tau \phi }\, dx dt \ge e^{2 \tau \Phi (r_2)} \Vert u\Vert _{L^2(\epsilon , T-\epsilon ; H^1(B_2 \setminus B_1))}^2. \end{aligned}$$

The inequalities (54)–(56) imply

$$\begin{aligned}&\Vert u\Vert _{L^2(\epsilon , T-\epsilon ; H^1(B_2))} \le e^{\tau } \left( \Vert L u\Vert _{L^2((0,T) \times B_4)} + \Vert u\Vert _{L^2(0,T; H^1(B_1))} \right) \\&\quad + e^{-p \tau } \Vert u\Vert _{L^2(0,T; H^1(B_4))}. \end{aligned}$$

where $p = \Phi (r_2) - \Phi (r_3) > 0$. The claim follows from [22, Lemma 5.2]. $\square$

### Proof of Theorem 1

We use notation from the proof of Theorem 7. By replacing $\omega$ with a smaller set we may assume without loss of generality that it is a ball of the above form $B_1$. We will show a local version of the claimed estimate where *B* is replaced by a ball of the form $B_2$. The general case follows by covering *B* by finite chains of balls starting from $\omega$, and by iterating the local result.

Let us first consider the case $u \in C^\infty (\mathbb R\times \Omega )$. We choose $0< r_0 < r_1$ and $r_2< r_3 < d(x_0, \Omega )$ and write $B_j = B(x_0, r_j)$, $j=0,3$. Let $0< \epsilon _0 < \epsilon$ and choose $\eta \in C^\infty (\mathbb R)$ such that $\eta = 0$ near the origin and $\eta (t) = 1$ for $t > \epsilon _0$. Let *w* be the solution of

$$\begin{aligned} L w&= \eta L u\quad \text { in } (0,T) \times B_3,\\ w|_{x \in \partial B_3}&= 0, \quad w|_{t=0} = 0, \end{aligned}$$

and set $v = u - w$. Note that $w \in C^\infty ([\epsilon _0, T] \times \Omega )$. Theorem 7 with *u* replaced by *v*, $B_1$ by $B_0$, $\Omega$ by $B_3$, and $t=0$ by $t=\epsilon _0$ implies that

$$\begin{aligned}&\Vert v\Vert _{L^2(\epsilon , T - \epsilon ; H^1(B_2))} \le C \Vert v\Vert _{L^2(\epsilon _0, T; H^1(B_0))} ^\kappa \Vert v\Vert _{L^2(\epsilon _0, T;H^1(B_3))}^{1-\kappa } \\ {}&\quad \le C (\Vert u\Vert _{L^2(\epsilon _0, T; H^1(B_0))} + \Vert L u\Vert _{0,-1})^\kappa (\Vert u\Vert _{L^2(\epsilon _0, T;H^1(B_3))} + \Vert L u\Vert _{0,-1})^{1-\kappa }. \end{aligned}$$

Here we have applied the energy estimate for the heat equation to *w*, see (57) below. Moreover,

$$\begin{aligned} \Vert u\Vert _{L^2(\epsilon , T-\epsilon ; H^1(B_2))}&\le \Vert v\Vert _{L^2(\epsilon , T-\epsilon ; H^1(B_2))} + C\Vert L u\Vert _{0,-1}. \end{aligned}$$

We choose $\chi \in C_0^\infty ((0,T) \times B_1)$ such that $\chi = 1$ in $(\epsilon _0, T-\epsilon _0) \times B_0$. Then $\chi u$ satisfies

$$\begin{aligned} L (\chi u) = \chi L u + [\chi , L] u, \quad (\chi u)|_{x \in \partial B_1} = 0, \quad (\chi u)|_{t=0} = 0, \end{aligned}$$

and as the commutator $[\chi , L]$ is of first order in space and zeroth order in time,

$$\begin{aligned} \Vert u\Vert _{L^2(\epsilon _0, T-\epsilon _0; H^1(B_0))} \le \Vert \chi u\Vert _{L^2(0,T; H^1(B_1))} \le C \Vert L u\Vert _{0,-1} + C \Vert u\Vert _{L^2((0,T) \times B_1)}. \end{aligned}$$

Analogously,

$$\begin{aligned} \Vert u\Vert _{L^2(\epsilon _0, T-\epsilon _0; H^1(B_3))} \le C \Vert L u\Vert _{0,-1} + C \Vert u\Vert _{L^2((0,T) \times \Omega )}. \end{aligned}$$

and we have shown the local estimate in the case that *u* is smooth. To conclude we observe that smooth functions are dense in the space (2). $\square$

Let us now turn to Theorem 2. In the case of convex, polygonal $\Omega$, the following simple lemma gives a weight function satisfying Condition 1.1 of [14].

### Lemma 7

Suppose that $\Omega \subset \mathbb R^n$ is open and convex set and that $\partial \Omega$ is piecewise smooth. Let $x_0 \in \Omega$ and $\epsilon > 0$ satisfy $\overline{B(x_0, \epsilon )} \subset \Omega$. Then there is $\rho \in C^\infty (\Omega )$ satisfying $\rho < 0$ in $\Omega$, $|\nabla \rho | = 1$ in $\Omega \setminus B(x_0, \epsilon )$ and $\partial _\nu \rho \le 0$ on $\partial \Omega$.

### Proof

We choose a strictly negative $\rho \in C^\infty (\Omega )$ such that $\rho (x) = -d(x,x_0)$ outside $B(x_0,\epsilon )$. Then $\nabla \rho = -(x-x_0)/|x-x_0|$ outside $B(x_0,\epsilon )$. By convexity, $(x - x_0) \cdot \nu \ge 0$ on $\partial \Omega$, and therefore $\partial _\nu \rho \le 0$ on the boundary. $\square$

Now [14, Lemma 1.3] can be written in the following form:

### Lemma 8

(Global Carleman estimate by Imanuvilov) Let $\Omega \subset \mathbb R^n$, $x_0 \in \Omega$ and $\epsilon > 0$ be as in Lemma 7. Let $\rho$ be the function given by Lemma 7. Let $T > 0$ and define $\ell = \lambda \hbar ^{-1} (e^{\alpha \rho } - 1)$, where $\hbar = t(T-t)$, $\alpha , \lambda > 0$. Then there are $C, \alpha , \lambda _0 > 0$ such that for all $w \in C^2([0,T] \times \overline{\Omega })$ satisfying $w = 0$ on $(0,T) \times \partial \Omega$ and all $\lambda > \lambda _0$ it holds that

$$\begin{aligned}&\int _0^{T} \int _{\Omega } \left( \tau |\nabla w|^2 + \tau ^3 |w|^2 \right) e^{2 \ell } dxdt\\&\quad \le C \int _0^{T} \int _\Omega |L w|^2 e^{2\ell } dxdt + C \int _0^{T} \int _{B(x_0,\epsilon )} \tau ^3 |w|^2 e^{2 \ell } dxdt. \end{aligned}$$

where $$\tau = \lambda \hbar ^{-1}$$.

### Proof of Theorem 2

We write $\mathcal C = C^\infty (0,T; C_0^\infty (\Omega ))$ and denote by $\mathcal H$ the energy space (4). Recall that $\mathcal H$ is continuously embedded in $C(0,T;L^2(\Omega ))$, see e.g. [24, Lemma 11.4], and that $\mathcal C$ is dense in $\mathcal H$. By density it is enough to consider the case $u \in \mathcal C$. Recall also the energy estimate for $v \in \mathcal H$, see e.g. [24, Theorem 11.3],

57 $$\begin{aligned} \Vert v\Vert _{\mathcal H} \le C \Vert v(0)\Vert _{L^2(\Omega )} + C \Vert L v\Vert _{L^2(0,T;H^{-1}(\Omega ))}. \end{aligned}$$

We write $f = \partial _t - \Delta u$, let $v \in \mathcal H$ be the solution of

$$\begin{aligned} \partial _t v - \Delta v = f, \quad v|_{t=0} = 0, \end{aligned}$$

and define $w = u - v$. Then *w* satisfies $\partial _t w - \Delta w = 0$. Choose $x_0 \in \omega$ and $\epsilon > 0$ such that $B(x_0, \epsilon ) \subset \omega$, and apply Lemma 8 on *w*. Note that $\tau ^3 e^{2 \ell } \rightarrow 0$ as $t \rightarrow 0$ or $t \rightarrow T$. Let $0< \delta _0 < \delta$, then $e^{2 \ell }$ is strictly positive on $[\delta _0, T-\delta _0] \times \Omega$. In particular,

$$\begin{aligned} \Vert w\Vert _{L^2((\delta _0,T-\delta _0) \times \Omega )} \le C \Vert w\Vert _{L^2((0,T) \times \omega )}. \end{aligned}$$

The estimate (57) implies that

$$\begin{aligned} \Vert w(\delta )\Vert _{L^2(\Omega )}^2 \le C \Vert w(s)\Vert _{L^2(\Omega )}^2, \quad s \in (0,\delta ). \end{aligned}$$

We integrate this over the interval $(\delta _0,\delta )$ to obtain

$$\begin{aligned} (\delta -\delta _0) \Vert w(\delta )\Vert _{L^2(\Omega )}^2 \le C \Vert w\Vert _{L^2((\delta _0,T-\delta _0) \times \Omega )}^2. \end{aligned}$$

By using (57) again, we have

$$\begin{aligned} \Vert v(\delta )\Vert _{L^2(\Omega )} + \Vert v\Vert _{L^2((0,T) \times \Omega )} \le C \Vert f\Vert _{L^2(0,T; H^{-1}(\Omega ))}. \end{aligned}$$

Hence

$$\begin{aligned} \Vert u(\delta )\Vert _{L^2(\Omega )}&\le C \Vert w\Vert _{L^2((0,T) \times \omega )} + C \Vert f\Vert _{L^2(0,T; H^{-1}(\Omega ))}\\&\le C \Vert u\Vert _{L^2((0,T) \times \omega )} + C \Vert f\Vert _{L^2(0,T; H^{-1}(\Omega ))}. \end{aligned}$$

The claim follows by applying (57) once more. $\square$
